# Hyaluronic acid 35 normalizes TLR4 signaling in Kupffer cells from ethanol-fed rats via regulation of microRNA291b and its target Tollip

**DOI:** 10.1038/s41598-017-15760-4

**Published:** 2017-11-15

**Authors:** Paramananda Saikia, Sanjoy Roychowdhury, Damien Bellos, Katherine A. Pollard, Megan R. McMullen, Rebecca L. McCullough, Arthur J. McCullough, Pierre Gholam, Carol de la Motte, Laura E. Nagy

**Affiliations:** 1 Center for Liver Disease Research, Department of Pathobiology, Cleveland, OH USA; 2 Departments of Gastroenterology and Hepatology, Cleveland Clinic, Cleveland, OH USA; 3 Department of Molecular Medicine, Case Western Reserve University, Cleveland, OH USA; 4 Department of Gastroenterology and Hepatology, University Hospital, Cleveland, OH USA

**Keywords:** Signal transduction, Alcoholic liver disease

## Abstract

TLR4 signaling in hepatic macrophages is increased after chronic ethanol feeding. Treatment of hepatic macrophages after chronic ethanol feeding with small-specific sized hyaluronic acid 35 (HA35) normalizes TLR4 signaling; however, the mechanisms for HA35 action are not completely understood. Here we used Next Generation Sequencing of microRNAs to identify negative regulators of TLR4 signaling reciprocally modulated by ethanol and HA35 in hepatic macrophages. Eleven microRNAs were up-regulated by ethanol; only 4 microRNAs, including miR291b, were decreased by HA35. Bioinformatics analysis identified Tollip, a negative regulator of TLR4, as a target of miR291b. Tollip expression was decreased in hepatic macrophages from ethanol-fed rats, but treatment with HA35 or transfection with a miR291b hairpin inhibitor restored Tollip expression and normalized TLR4-stimulated TNFα expression. In peripheral blood monocytes isolated from patients with alcoholic hepatitis, expression of TNFα mRNA was robustly increased in response to challenge with lipopolysaccharide. Importantly, pre-treatment with HA35 reduced TNFα expression by more than 50%. Taken together, we have identified miR291b as a critical miRNA up-regulated by ethanol. Normalization of the miR291b → Tollip pathway by HA35 ameliorated ethanol-induced sensitization of TLR4 signaling in macrophages/monocytes, suggesting that HA35 may be a novel therapeutic agent in the treatment of ALD.

## Introduction

Alcoholic liver disease (ALD) develops in approximately 20% of all alcoholics with a higher prevalence in females^1^. The development of fibrosis and cirrhosis is a complex process involving both parenchymal and non-parenchymal cells resident in the liver, as well as the recruitment of other cell types to the liver in response to damage and inflammation^2^. There is a growing appreciation that inter-organ cross talk between the intestine and liver drives the progression of ethanol-induced liver injury. Impairment of intestinal barrier function is associated with the progression of ethanol-induced liver injury in both humans after heavy alcohol consumption and in rodent models of chronic ethanol exposure^3^. Increased exposure of Kupffer cells, the resident hepatic macrophages, to gut-derived LPS during chronic ethanol, activates TLR4-dependent production of inflammatory mediators^4^. In addition to causing increased contact with LPS, we and others have shown that chronic ethanol exposure also sensitizes Kupffer cells to LPS, resulting in increased production of inflammatory mediators^4^. Further, dysregulation of the microbiome during chronic ethanol exposure likely contributes to the development of liver disease^5^. Despite this understanding of the progression of ALD, effective prevention and treatment strategies have remained elusive.

Hyaluronan (HA), a large linear glycan, is an abundant extracellular matrix component and is produced as a straight chain polymer strictly composed of repeating disaccharides of D-glucuronic acid and N-acetylglucosamine. HA is produced at cell surfaces by one or more plasma membrane hyaluronan synthases (HAS1, HAS2 and HAS3)^6^. High molecular weight matrix hyaluronan can be degraded in response to injury^7^. The resulting HA fragments can have either pro-inflammatory or protective, anti-inflammatory effects, depending on the size and molecular structure of the HA fragments, as well as the cellular and tissue environment^7^.

Recently, a small specific-sized hyaluronic acid with an average molecular weight of 35kD (HA35) was identified as a potential therapeutic agent for ALD^8^. HA35 normalized TLR4-stimulated cytokine production in Kupffer cells and protected mice from short-term ethanol-induced gut and liver injury^8^. In order to understand the mechanisms for the anti-inflammatory effects of HA35 on Kupffer cells after chronic ethanol feeding, an un-biased Next Generation Sequencing analysis of miRNAs was performed. Focusing on miRNAs whose expression is decreased by ethanol revealed that chronic ethanol enhanced a miR181b-3p → importin α5 regulatory axis in hepatic macrophages that contributed to the sensitization of TLR4-dependent cytokine expression in these Kupffer cells. Treatment with HA35 normalized this pathway and reduced inflammatory activity^8^.

In this investigation, we have taken the complementary approach to understanding the anti-inflammatory mechanisms for HA35 action in Kupffer cells after chronic ethanol exposure and analyzed miRNAs whose expression was increased in Kupffer cells after chronic ethanol feeding and in turn restored by *ex vivo* treatment of HA35. Chronic ethanol feeding decreased the expression of miR291b in Kupffer cells and treatment with HA35 restored expression via a CD44-dependent mechanism. Importantly, Tollip, a negative regulator of TLR4-MyD88-IRAK signaling^9,10^, was identified as a target of miR291b. Tollip is known to be post-transcriptionally regulated by other miRNAs, including miR-31^11^. Tollip suppresses the sensitivity of macrophages to stimulation by LPS, as well as IL1-β^9^, although this response may vary with dose of LPS^12,13^; Tollip expression was decreased in Kupffer cells after chronic ethanol, but restored by HA35; restoration of Tollip expression contributed to the normalization of TLR4 signaling in Kupffer cells after chronic ethanol feeding.

## Results

### HA35 decreased LPS and PamCys3K-stimulated TNFα mRNA expression in primary cultures of Kupffer cells from ethanol and pair-fed rats, as well as in PBMCs from patients with alcoholic hepatitis

**Figure 1:**
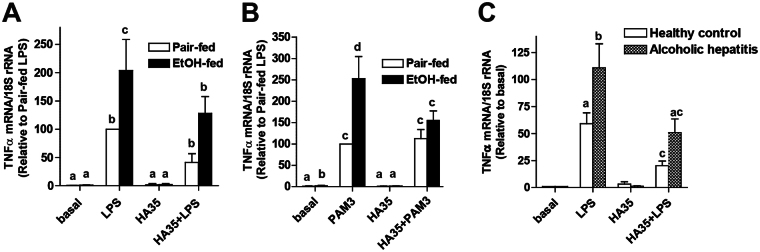
HA35 decreased LPS and PamCys3K-stimulated TNFα mRNA expression in primary cultures of rat Kupffer cells from ethanol and pair-fed rats, as well as PBMCs from patients with alcoholic hepatitis. Wistar rats were allowed free access to a Lieber-DeCarli ethanol diet or pair-fed control diet for 4 weeks. Kupffer cells were isolated and cultured overnight. Kupffer cells were then treated with 100 μg/ml HA35 for 5 h and then challenged with (**A**) 10 ng/ml LPS or (**B**) 10 ng/ml PamCys3K (PAM3) for an additional 1hr. Expression of TNFα mRNA was measured by qRT-PCR and normalized to 18 S rRNA. (**C**) PBMCs isolated from patients with AH and healthy controls, cultured overnight and then treated or not with 100 μg/ml HA35. PBMCs were then challenged with 10 ng/ml LPS for an additional 1hr. Expression of TNFα mRNA was measured by qRT-PCR and normalized to 18 S rRNA. (**A**/**B**) n = 4; (**C**) n = 10 for AH and 6 for healthy controls. Values represent means ± SEM. Values with different alphabetical superscripts are significantly different from each other, p < 0.05.

Kupffer cell activation is an early event during progression of ALD and chronic ethanol exposure sensitizes Kupffer cells to TLR4-dependent cytokine production. Understanding the molecular mechanisms for this sensitization and identification of anti-inflammatory agents that can restore normal responses is a clinically relevant area of investigation. We have recently discovered that small-specific-sized HA35 can normalize TLR4-mediated cytokine expression by rat Kupffer cells after chronic ethanol exposure^8^ (Fig. 1A). Chronic ethanol also sensitized Kupffer cells to stimulation by PamCys3K, a TLR2 ligand, when compared to the response of Kupffer cells isolated from pair-fed control rats (Fig. 1B). Pre-treatment with HA35 also normalized the sensitivity of Kupffer cells from ethanol-fed rats to stimulation by PamCys3K, without affecting the sensitivity of cells from pair-fed controls (Fig. 1B).

In order to determine whether HA35 regulates TLR4-dependent cytokine expression in patients with alcoholic hepatitis (AH), we studied isolated peripheral blood monocytes (PBMCs) from a cohort of patients with moderate to severe AH, with Model for End Stage Liver Disease (MELD) scores ranging from 17–33, as well as a cohort of healthy control subjects. Table 1 provides information on the clinical characteristics of the AH patients. PBMCs isolated from patients with AH or healthy controls were cultured with or without HA35 for 5 h and then challenged with LPS (Fig. 1C). LPS increased the expression of TNFα mRNA in PBMCs from both controls and patients with AH; the LPS-stimulated response was greater in patients with AH compared to controls, as has been previously reported^14,15^. LPS-stimulated expression of TNFα mRNA was reduced in PBMCs from both patients with AH and healthy controls that were pre-treated with HA35 (Fig. 1C).

**Table 1:** Clinical characteristics of alcoholic hepatitis patients and healthy controls. Values are median and intraquartile range, n = 10 for alcoholic hepatitis and n = 6 for healthy controls.

Characteristics	Alcoholic hepatitis patients	Healthy controls
	Median (25–75 IQR)	Median (25–75 IQR)
Age (years)	47(26–60)	27(23–48)
Male n (%)	60(6)	50(3)
Alcohol Consumption (g/day)	60(37–100)	30(12.5–50)
**Laboratory and hemodynamic parameters**		
Hemoglobin (g/dL)	9.7(7.9–12.4)	ND
Leukocyte count × 10^9^/L	9.2 (4.2–18.4)	ND
Platelet count × 10^9^/L	148.8 (69–329)	ND
AST (U/L)	105 (50–168)	ND
ALT (U/L)	42.4 (17–69)	ND
Serum albumin (g/dL)	2.7 (1.9–3.5)	ND
Serum creatinine (mg/dL)	1.1 (0.48–1.5)	ND
Serum bilirubin(mg/dL)	12.4 (2.2–27.2)	ND
MELD score	21.6 (17–33)	ND
Discriminant Function Scores	42.72 (3.1–64.7)	ND

### siRNA knock-down of receptors for HA in Kupffer cells from ethanol-fed rats demonstrated the importance of CD44 in normalizing TLR4-dependent TNFα expression

**Figure 2:**
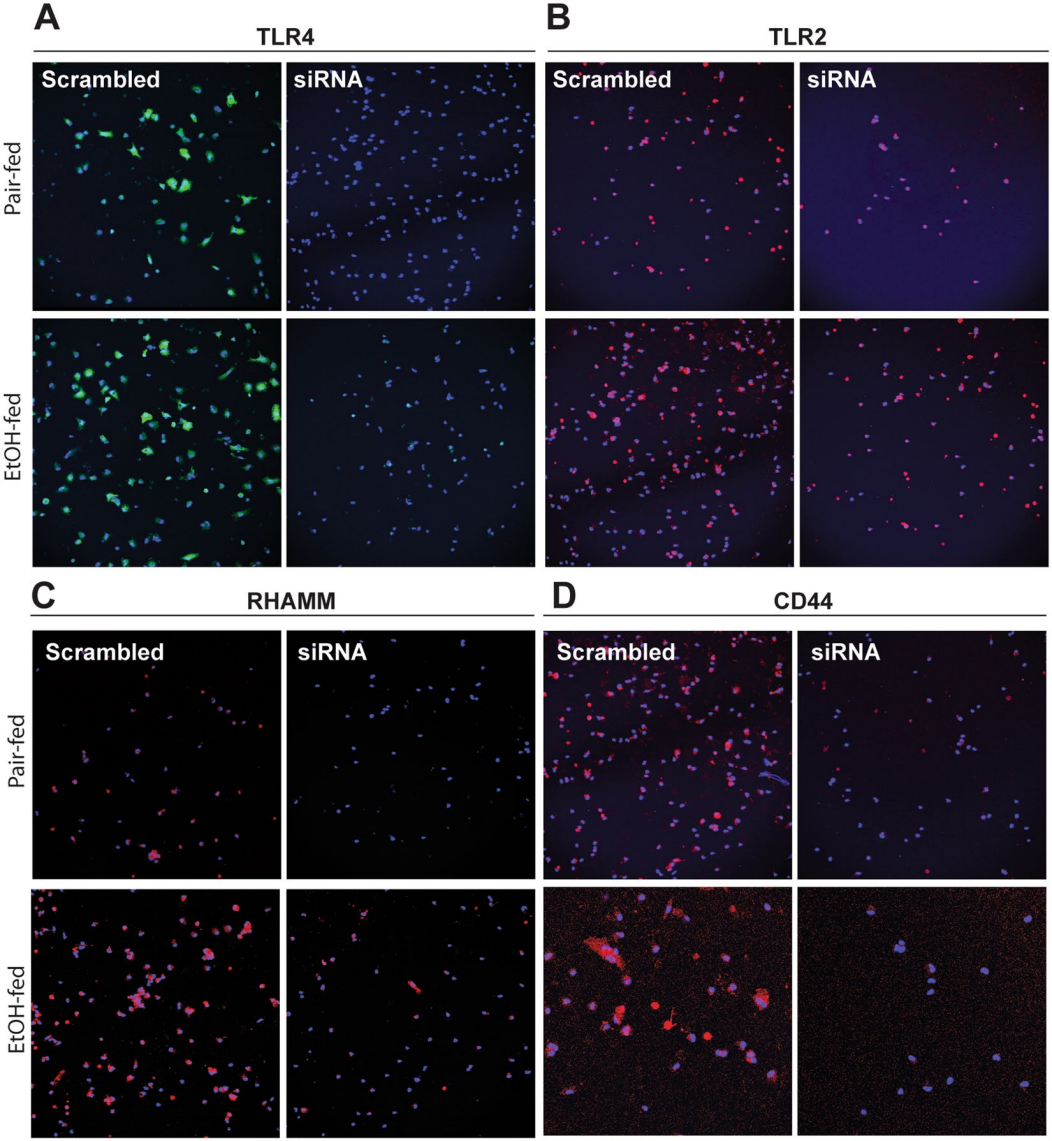
siRNA knockdown of HA receptors in Kupffer cells from pair- and ethanol-fed rats. Wistar rats were allowed free access to a Lieber-DeCarli ethanol diet or pair-fed control diet for 4 weeks. Kupffer cells were isolated and nucleofected with 25 nM of scrambled siRNA or siRNA targeted to **(A)** TLR4, **(B)** TLR2, **(C)** RHAMM and **(D)** CD44. 24 h post-nucleofection, expression of **(A)** TLR4 (green), **(B)** TLR2 (red), **(C)** RHAMM (red) and **(D)** CD44 (red) was visualized by immunostaining with specific antibodies. Nuclei were labelled with DAPI (blue). All images were acquired using 20x objectives and are representative of 3 independent experiments.

HA binds to different cell surface receptors to initiate cell signaling. CD44, TLR4, TLR2, and RHAMM are known to interact with HA; ligation of these receptors by HA depends both on the size of the HA molecule and the specific cell type^16–19^. In order to identify the key receptor(s) mediating the protective effect of HA35 in Kupffer cells, expression of each of these four receptors was knocked-down by nucleofection of siRNA targeted to each receptor. Comparison in the expression of TLR4 (Fig. 2A), TLR2 (Fig. 2B), RHAMM (Fig. 2C) and CD44 (Fig. 2D) between cells transfected with scrambled versus targeted siRNA confirmed effective knock-down of each of these receptors.

**Figure 3:**
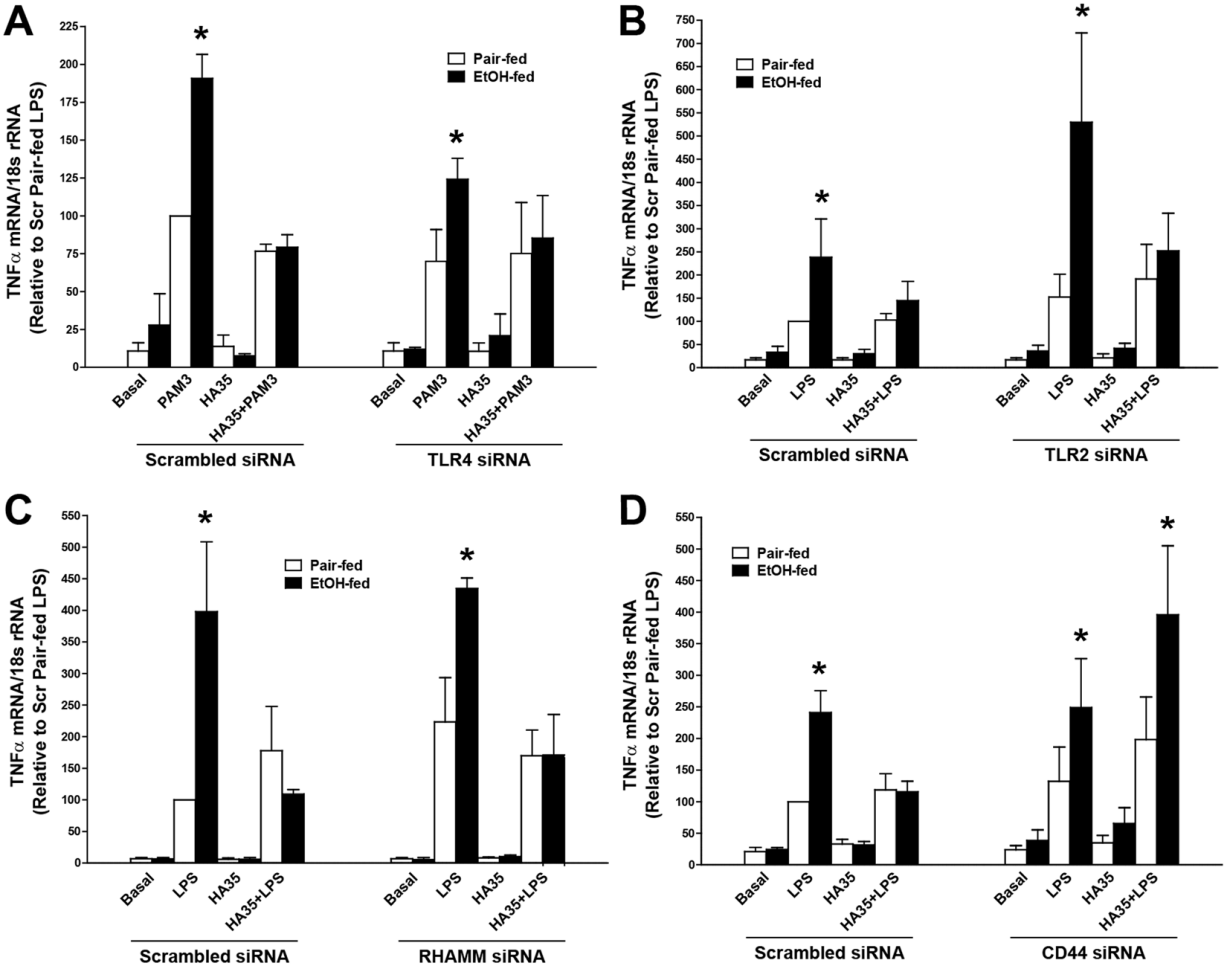
siRNA knockdown of HA receptors in Kupffer cells from pair- and ethanol-fed rats. Wistar rats were allowed free access to a Lieber-DeCarli ethanol diet or pair-fed control diet for 4 weeks. Kupffer cells were isolated and nucleofected with 25 nM of scrambled siRNA or siRNA targeted to **(A)** TLR4, **(B)** TLR2, **(C)** RHAMM and **(D)** CD44. 18 h post-nucleofection, Kupffer cells were treated with or without 100 µg/ml HA35 for 5 h and then challenged with 10 ng/ml **(A)** PamCys3K or (**B**,**C**,**D**) LPS for 1hr and TNFα mRNA measured by qRT-PCR and normalized to 18 S rRNA. Values represent means ± SEM, n = 4, *p < 0.05 compared to pair-fed within a treatment group.

When TLR4 was knocked-down in primary cultures of Kupffer cells with targeted siRNA, LPS stimulation of TNFα expression was very low, as would be expected (Supplemental Fig. 1). Therefore, in order to ascertain the contribution of TLR4 to the HA-mediated normalization of Kupffer cell cytokine expression, after knock-down of TLR4, Kupffer cells were challenged with the TLR2 ligand PamCys3K (Fig. 3A). Challenge with PamCys3K increased expression of TNFα in Kupffer cells nucleofected with either scrambled siRNA or siRNA targeted to knock-down TLR4 (Fig. 3A). However, even when TLR4 was knocked-down, pre-treatment with HA35 still reduced PamCys3K-stimulated expression of TNFα mRNA (Fig. 3A). Similarly, when Kupffer cells were nucleofected with siRNA targeting TLR2 and RHAMM, HA35 pre-treatment was still reduced LPS-stimulated TNFα expression (Fig. 3B/C). Only when the expression of CD44 was knocked-down was the protective effect of HA35 on LPS-stimulated TNFα expression ameliorated (Fig. 3D). Taken together, these data indicate that CD44 mediates the effects of HA35 on Kupffer cells from ethanol-fed rats.

### Identification of multiple miRNAs up-regulated by ethanol and down-regulated by HA35 in Kupffer cells by Next Generation Sequencing

**Figure 4:**
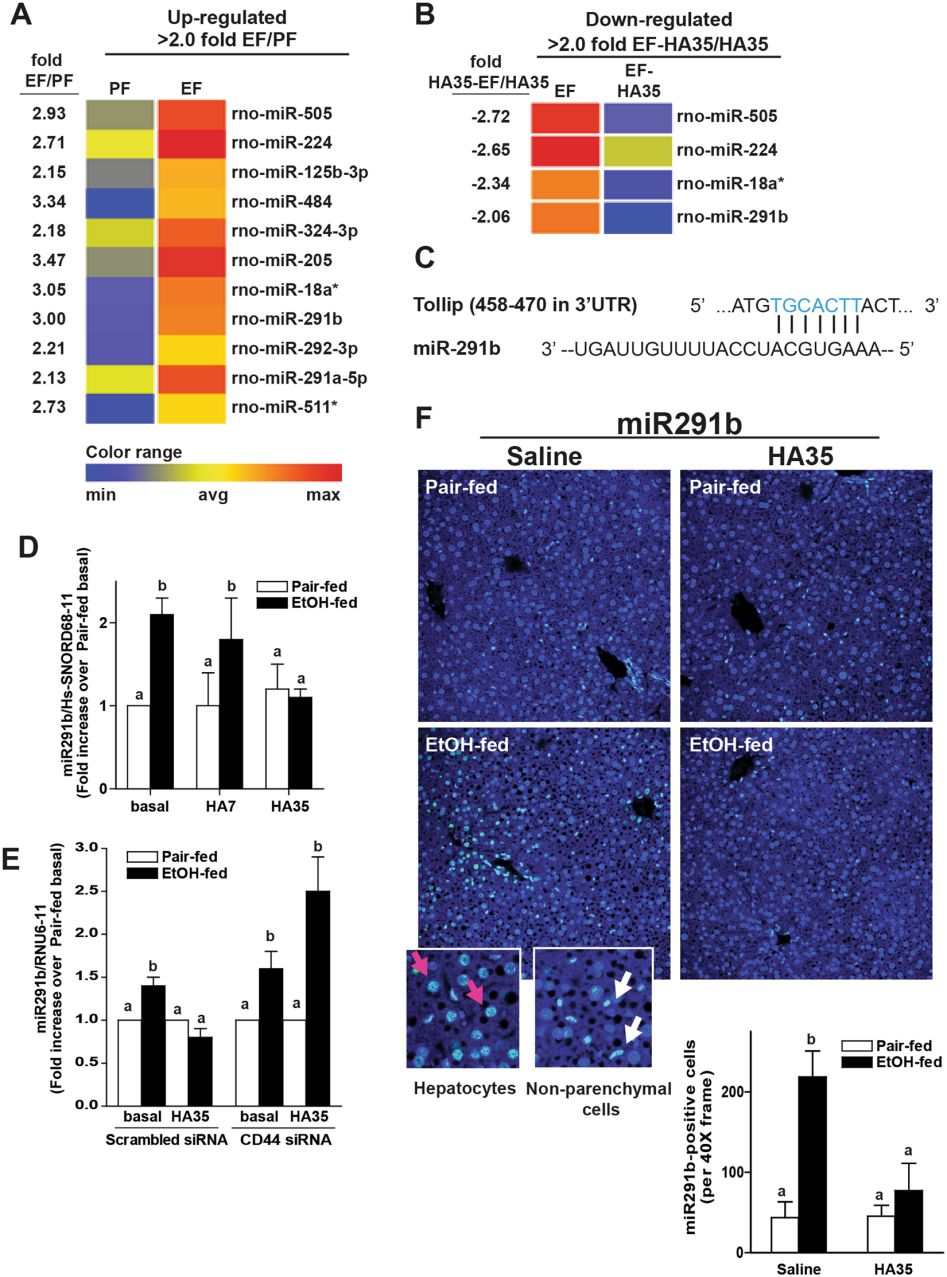
Next Generation Sequencing of miRNAs in Kupffer cells from ethanol- and pair-fed rats after treatment with or without HA35. (**A–D**) Wistar rats were allowed free access to a Lieber-DeCarli ethanol diet or pair-fed control diet for 4 weeks. Kupffer cells were isolated and cultured overnight. Kupffer cells were then treated with or without 100 μg/ml HA35 for 5 h. Total miRNAs were isolated and analyzed by NGS. (**A**) Heat maps illustrate the changes in expression for the 11 miRNAs identified whose expression was increased by more than 2-fold by ethanol feeding. (**B**) Of these eleven miRNA, heat maps are shown for the 4 miRNAs whose expression was decreased by treatment with HA35. Fold changes are shown in the column to the left of the heat maps. (**C**) Predicted binding site for miR291b in the 3′UTR of Tollip (miRDB^22^). Sequence alignment between miR291b and Tollip is illustrated. (**D**) Kupffer cells isolated from ethanol- and pair-fed rats were treated or not with HA35 or HA7 for 5 h and expression of miR291b measured by qRT-PCR and normalized to Hs-SNORD68-11. (**E**) Isolated Kupffer cells were nucleofected with scrambled siRNA or siRNA targeted against CD44. 18 h post-nucleofection, Kupffer cells were treated or not with 100 μg/ml HA35 for 5 h and expression of miR291b measured by qRT-PCR and normalized to Hs-SNORD68-11. (**F**) C57BL/6 J mice were allowed free access to an ethanol containing diet for 4 days (2 days at 1% (v/v) ethanol, followed by 2 days 6% (v/v) ethanol) or pair-fed an isocaloric control diet. Mice were gavaged once daily for the last 3 days of ethanol feeding with HA35 at 15 mg/kg body weight or saline as a vehicle control the last three days of the study. Paraffin-embedded livers were de-paraffinized followed by *in situ* hybridization for miR291b using locked nucleic acid probes tagged with digoxigenin at both the 5′and 3′ ends. All images were captured using 40X objectives. miR291b –positive cells were counted using Image Pro-Plus software and analyzed. Zoomed images illustrate positive staining in cells identified by morphology as hepatocytes (pink) and non-parenchymal cells (white). (**A**,**B**) n = 3 (**C**) n = 5 (**D**) n = 4 (**E**) n = 3–6. (**C**,**D**,**E**) Values represent means ± SEM. Values with different alphabetical superscripts are significantly different from each other, p < 0.05.

miRNAs, non-protein coding RNAs of 20–22 nucleotides, are key regulators of inflammatory responses in innate immune cells, providing both positive and negative regulation of TLR4 signaling^20,21^. Therefore, we hypothesized the regulation of miRNA expression in Kupffer cells contributed to their response to ethanol and HA35. To test this hypothesis, Next Generation Sequencing (NGS) of miRNAs isolated from Kupffer cells from pair- and ethanol-fed rats after treatment with or without HA35 for 5 h was conducted on the Illumina platform. Ethanol feeding up-regulated 11 miRNAs by more than two-fold (Fig. 4A). Of these miRNAs, HA35 treatment normalized the expression of only 4 miRNAs, including miR291b (Fig. 4B). Using the miRDB (www.mirdb.org) analysis tool^22^, ~350 miR291b-targeted genes were retrieved with a target prediction score greater than 60; predicted target scores greater than 60 indicate a high likelihood of miRNA binding to a particular target (Supplemental Table 1). Tollip, a negative regulator of the MyD88-dependent pathway of TLR2 and TLR4 signaling, was identified to have a binding site in its 3′UTR that recognizes miR291b (Fig. 4C) with a target prediction score of 79 in both rat and mouse. Further, miR291b was ranked at 15 from 45 miRNAs predicted to bind to Tollip in rat and 11 from 92 miRNAs predicted to bind Tollip in mouse. Target Scan (www.targetscan.org), another bioinformatics tool, also confirmed Tollip as a potential target of miR291b. Based on these *in silico* analyses, we predicted that increased expression of miR291b in response to ethanol feeding would decrease the expression of Tollip protein. If miR291b regulates the expression of Tollip, we hypothesized that it could be a critical mediator of both the sensitization of Kupffer cells from ethanol-fed rats to TLR2/TLR4 signaling, as well as the protective effects of HA35.

The reciprocal regulation of miR291b by ethanol and HA35 was confirmed by real-time PCR (Fig. 4D). Expression of miR291b was not sensitive to pre-treatment with HA7, a small-specific sized HA fragment that does not normalize TLR4 signaling in Kupffer cells from ethanol-fed rats^8^ (Fig. 4D). In order to confirm a role for CD44 in mediating the impact of HA35 on expression of miR291b, Kupffer cells were nucleofected with scrambled siRNA or siRNA targeting CD44. Similar to non-transfected cells, when cells were nucleofected with scrambled siRNA, miR291b expression was increased in Kupffer cells from ethanol-fed rats and normalized by treatment with HA35 (Fig. 4E). However, when CD44 was knocked-down in Kupffer cells, expression of miR291b was not decreased by HA35 treatment (Fig. 4E).

We have recently reported that treatment of mice with HA35 by gavage protects mice from short-term ethanol-induced liver and gut injury^8^. Here we investigated whether short-term ethanol feeding and/or oral provision of HA35 affected the expression of hepatic miR291b. Short-term ethanol feeding increased expression of miR291b in liver; both hepatocytes and non-parenchymal cells exhibited expression as assessed via *in situ* hybridization (Fig. 4F). Treatment of mice with HA35 during ethanol feeding decreased expression of miR291b in both cell types (Fig. 4F).

### Expression of Tollip protein was lower in Kupffer cells from ethanol-fed rats and increased by HA35 or the presence of a miR291b hairpin inhibitor

**Figure 5:**
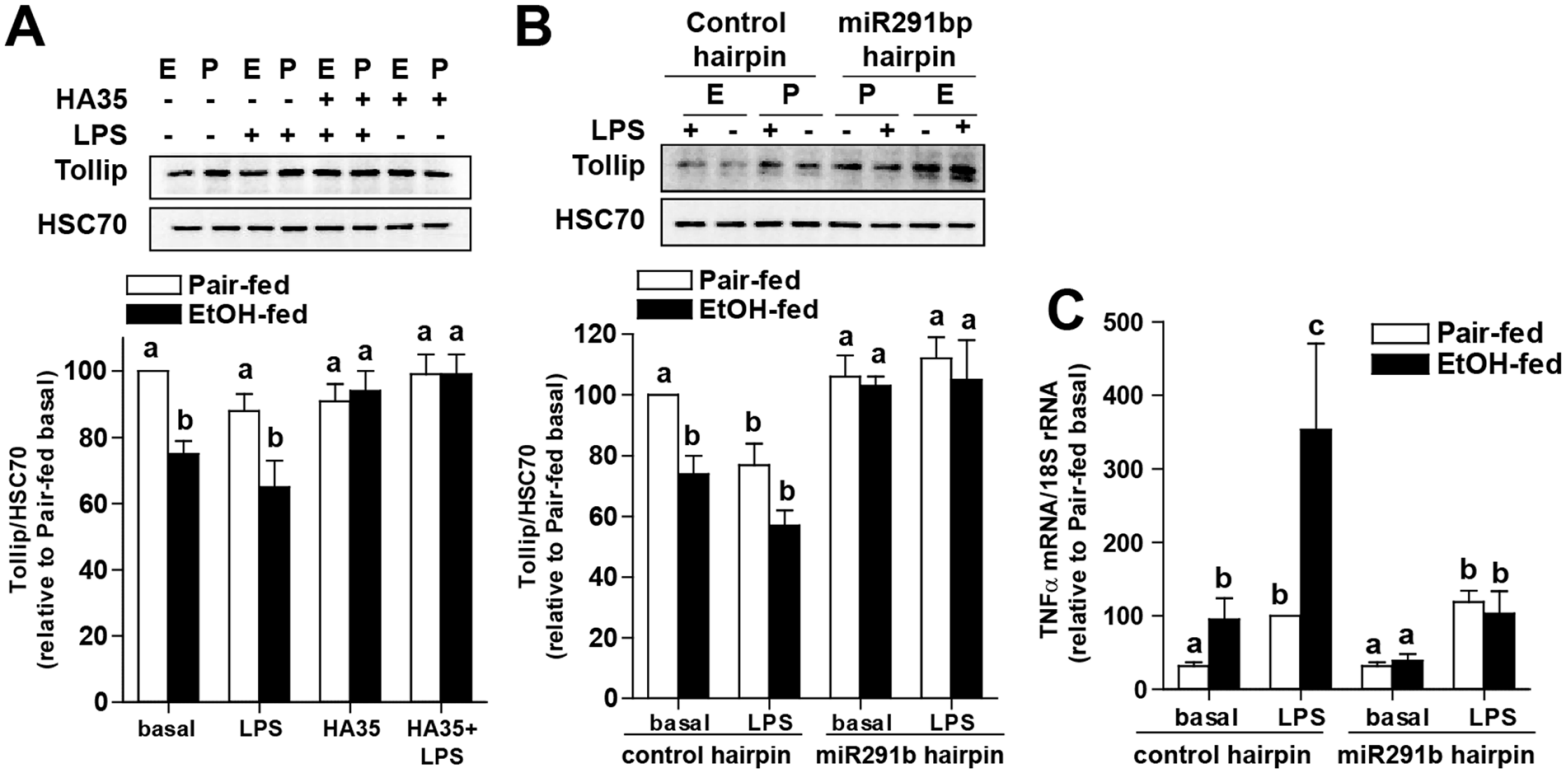
Regulation of Tollip expression by ethanol, HA35 and miR291b in primary cultures of Kupffer cells. (**A**) Kupffer cells isolated from ethanol- and pair-fed rats were cultured overnight and then treated with 100 µg/ml HA35 for 5 h and then challenged with 10 ng/ml LPS for an additional 60 min. **(B,C)** Kupffer cells isolated from ethanol- and pair-fed rats were nucleofected with either control hairpin or miR291b hairpin inhibitor 18 h post-nucleofection, Kupffer cells were treated or not with 100 µg/ml HA35 for 5 h and then challenged with 10 ng/ml LPS for an additional 1 hr. **(A,B)** Kupffer cells were lysed and Tollip expression measured by Western blot. HSC70 was used as a loading control. Images of the blots were cropped. Full size blots are shown in Supplemental Information Fig. 2. (**C**) Expression of TNFα mRNA was determined by qRT-PCR and normalized to 18 S. n = 4–5. Values represent means ± SEM. Values with different alphabetical superscripts are significantly different from each other, p < 0.05.

If increased expression of miR291b modulated Tollip expression, then we would expect to see less Tollip protein in Kupffer cells from ethanol-fed rats. Western blot analysis revealed that Tollip expression was lower in Kupffer cells from ethanol-fed rats; pre-treatment with HA35 normalized expression in Kupffer cells from ethanol-fed rats, but did not impact Tollip expression in Kupffer cells from pair-fed rats (Fig. 5A). Challenge with LPS for 60 min had no effect on Tollip protein quantity (Fig. 5A). If the HA35-mediated reduction in miR291b expression mediated the ethanol-induced increase in the quantity of Tollip protein, then transfection of a miR291b hairpin inhibitor should normalize Tollip protein, as well as TLR4 signaling, in Kupffer cells from ethanol-fed rats. Transfection with a miR291b hairpin inhibitor, but not with a control hairpin, increased Tollip protein expression (Fig. 5B) and normalized LPS-stimulated expression of TNFα mRNA (Fig. 5C).

## Discussion

**Figure 6:**
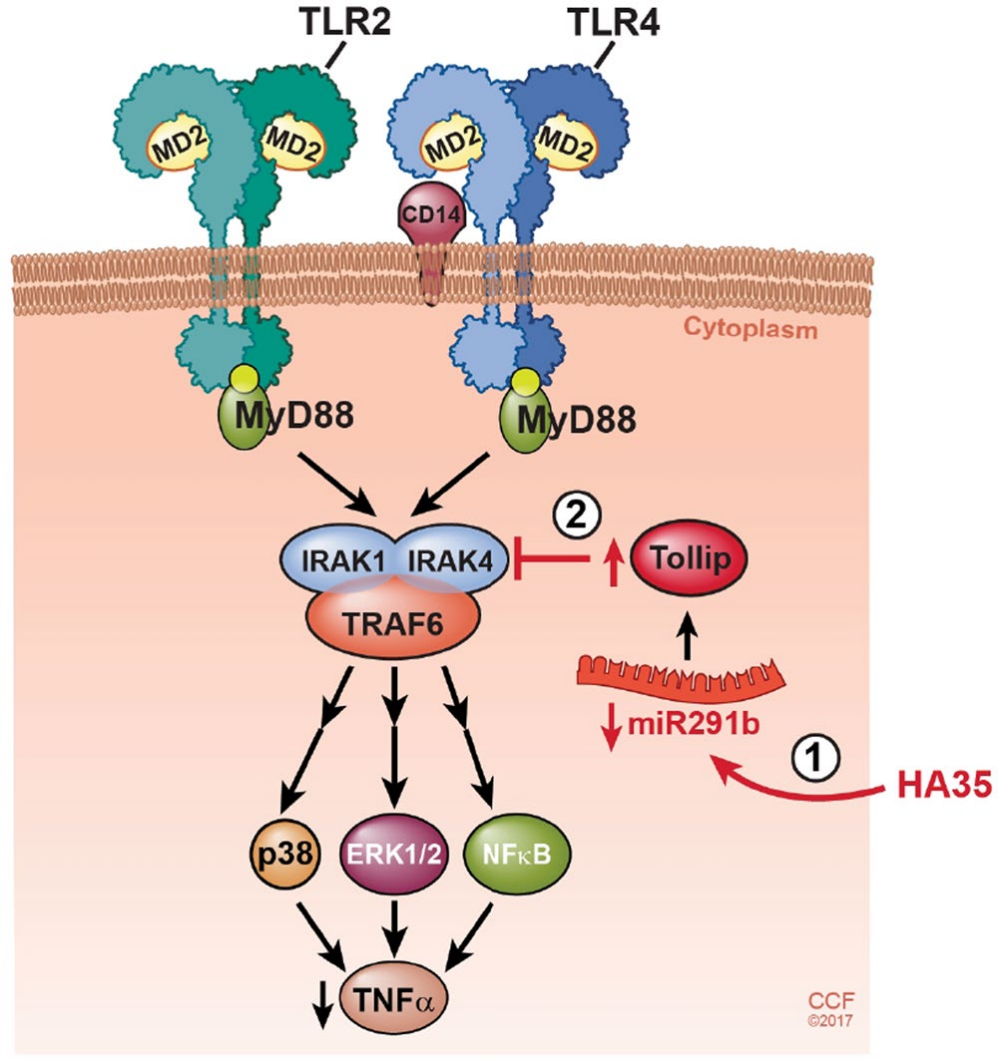
Model illustrating the interactions between HA35 on expression of miR291b and Tollip in Kupffer cells from ethanol-fed rats. Chronic ethanol feeding increases the expression of miR291b which in turn results in decreased expression of Tollip. This decreased expression of Tollip contributes to enhanced TNFα expression in response to activation of TLR2/TLR4. Treatment of Kupffer cells from ethanol-fed rats with HA35 *ex vivo* decreased the expression of miR291b and increased Tollip, reducing the sensitivity of Kupffer cells to TLR2/TLR4-mediated TNFα expression. Reprinted with permission, Cleveland Clinic Center for Medical Art & Photography© 2016. All Rights Reserved.

Understanding the mechanisms by which chronic ethanol sensitizes macrophages to TLR-dependent activation is critical to the development of effective therapeutics for treatment of alcoholic liver disease. An expanding number of miRNAs have been identified to regulate expression of the TLR2/4 signaling machinery, as well as the transcription factors and cytokines induced by TLR2/4 signaling^21^. Only a few examples of the impact of chronic ethanol on miRNA controlling TLR-mediated responses have been reported. For example, miR155 expression is up-regulated in Kupffer cells and contributes to ethanol-induced stabilization of TNFα mRNA^23^. Chronic ethanol feeding decreases the expression of miRNA181b-3p, which in turn upregulates the expression of the nuclear-cytoplasmic shuttling protein, importin α5, contributing to increased p65 translocation to the nucleus in Kupffer cells after chronic ethanol feeding^8^. Here we have identified miR291b as an additional miR up-regulated by chronic ethanol that contributes to sensitization of TLR2/4 signaling by decreasing the expression of Tollip, a negative regulator of MyD88-dependent signaling (Fig. 6). Expression of miR291b and Tollip, as well as TLR2/4-stimulated TNFα expression can be normalized by treatment of Kupffer cells with HA35. Importantly, treatment of PBMCs isolated from patients with AH with HA35 also potently reduced the sensitivity of TLR4-mediated cytokine expression.

The intracellular signaling pathways activated by TLRs are mediated through the adaptor molecules MyD88 and TRIF, ultimately leading to the induction of an inflammatory response^24^. Given the critical importance of TLR signaling in inflammation, it is not surprising that multiple negative regulators contribute to the modulation of the inflammatory response by dampening TLR signals. Important negative regulators include Tollip, as well as suppressor of cytokine signaling 1 (SOCS-1) and interleukin 1 receptor-associated kinase (IRAK)-M^25^. Tollip protein functions as a negative regulator of the TLR4 signaling pathway through its interaction with IRAK^10^. Tollip binds to IRAK-1 and halts signaling by blocking recruitment of IRAK-4 to the myddosome, thereby, preventing the phosphorylation and activation of IRAK-1^10^. Interestingly, Kupffer cells have a higher expression of Tollip mRNA compared to peripheral monocytes, likely contributing to their relatively tolerogenic status in the liver^26^. Here we find that chronic ethanol feeding decreases the expression of Tollip protein in Kupffer cells, consistent with the increased sensitivity of TLR2 and TLR4 signaling after chronic ethanol (Fig. 1). Tollip expression can be regulated at the transcriptional^27^, as well as post-transcriptional level^11^. The 3′UTR of Tollip contains a miR31 binding site; miR31 binding inhibits translation^11^. Here, using TargetScan, we identified a miR291b binding site in the Tollip 3′UTR that was associated with decreased Tollip protein quantity in Kupffer cells. Importantly, treatment of Kupffer cells from ethanol-fed rats with either HA35 or a miR291b inhibitor restored Tollip expression and normalized TLR4 sensitivity (Fig. 5). Taken together, these data indicate that extent of Tollip expression in Kupffer cells likely plays an important role as a rheostat of TLR2/4 signaling in the liver.

Expression of miR291b was also found to be increased in the liver of mice in response to short-term ethanol feeding (Fig. 4). Interestingly, miR291b was detected in both non-parenchymal cells and hepatocytes; treatment of mice with HA35 prevented these ethanol-induced increases in both cell types. While the increase in miR291b expression in non-parenchymal cells is consistent with our data from Kupffer cells after chronic ethanol feeding, induction of miR291b in hepatocytes may also be of pathophysiological significance, as miR291b-3p has been reported to induce hepatocyte apoptosis by downregulating HuR and Bcl-2 expression^28^ and also impact glucose and lipid homeostasis in hepatocytes^29,30^. Therefore, future investigations will investigate if expression of miR291b in hepatocytes in response to ethanol exposure might play an important role in ethanol-induced hepatocyte injury.

HA, a major glycosaminoglycan in the extracellular matrix, is recognized as an important danger associated molecular pattern (DAMP) that regulates innate immunity^7,31^. Despite the potent ability of HA to stimulate inflammatory responses^7^, specific-sized HA fragments can also have protective, anti-inflammatory effects^19^. Purified milk HA increases expression of the broad spectrum antimicrobial peptide, human β defensin 2 (HBD2)^31^ and protects from *Salmonella* infection in human epithelium *in vitro* ^32^. Highly purified HA35 specifically mimics the effects of milk HA, increasing expression of HBD2 and protecting epithelium from *Salmonella*. HA35 also protects mice from *in vivo* infection with *Citrobacter rodentium* (a model for human enteropathogenic *E. coli* infection) and modulates epithelial ZO1 expression *in vivo* in multiple models of colitis^33^. HA is being developed as a novel dietary supplement for formula-fed infants and children at risk for enteric bacterial infection, as well as patients who suffer from bacterial dysbiosis, e.g. individuals with inflammatory bowel disease (IBD). Our recent data indicate that HA35 can also protect mice from gut and liver injury in response to short-term ethanol feeding^8^.

The concentration of endogenous HA35 in healthy individuals and patients with liver disease is not known. However, it is unlikely that there is a sufficient concentration of endogenous HA35 to mimic the results of our pharmacological interventions with purified HA35. For example, it is estimated that the total hyaluronan content in a 70 kg human is 15 g^34^. The dose of HA35 provided therapeutically to mice in our study was 1.5 g/kg or the equivalent of 105 g in a 70 kg human. Further, total concentrations of all sizes of hyaluronan circulating in the serum, measured by ELISA, are on the order of 10–100 micrograms/L in healthy individuals^35^ and about ~50 micrograms/L in rats^36^. In different stages of alcoholic liver disease, total concentration of all sizes of hyaluronan circulating increases with severity of inflammation and fibrosis to concentrations ranging from 500–1000 micrograms/L^37,38^. Total HA is more predictive of stage of fibrosis, rather than severity of inflammation^37,38^. Viewed from the perspective of the circulating concentrations of all sizes of hyaluronan both in health and liver disease, it is unlikely that concentrations of endogenous specific-sized HA35 would likely have an impact on TLR4 signaling, as purified HA35 was used at a pharmacological concentration of 100 grams/L (100 micrograms/ml) in our cell based studies.

HA communicates with many cell types in a size-specific manner, using at least four signaling receptors including: (1) CD44, a ubiquitously-expressed cell surface protein that recognizes HA sizes greater than 8 sugar moieties in length^16^; (2) RHAMM (receptor for HA mediated motility) which is important in signaling cell migration^17^; (3) toll-like receptor (TLR) pattern recognition molecules TLR4 and TLR2^18,19^. Here we have identified CD44 as the critical receptor for HA35-mediated anti-inflammatory effects in Kupffer cells after chronic ethanol feeding (Fig. 3). The interaction of hyaluronan with CD44 is dependent on both the size of the HA and cellular variant of CD44; depending on the context HA ligation of CD44 can be either pro- or anti-inflammatory^39^. Interestingly, CD44 can also interact with other ligands, including osteopontin^40^. Under some conditions, osteopontin, like HA35, can protect mice from ethanol-induced liver injury^41^.

In summary, here we have identified a miR291b-mediated down-regulation of Tollip expression in Kupffer cells from ethanol-fed rats that contributes to the sensitization of TLR2/4 signaling after chronic ethanol (Fig. 6). Importantly, this dysregulation of TLR2/4 signaling was normalized by the treatment of Kupffer cells or PBMCs from patients with alcoholic hepatitis with HA35. These data add to a growing number of miRNAs involved in the response to macrophages to ethanol, including miR155^23^ and miR181b-3p^8^. Through normalization of expression miR291b, as well as miR181b-3p^8^, treatment of HA35 protected from the effects of ethanol in Kupffer cells and PBMCs from patients with AH, suggesting that HA35 has potential as a therapeutic agent for the treatment of ALD.

## Materials and Methods

### Chronic ethanol feeding to rats

All procedures involving animals were approved by and carried out according to the guidelines of the Institutional Animal Care and Use Committee at the Cleveland Clinic. Adult male Wistar rats weighing 140–150 g were purchased from Envigo (Indianapolis, IN). Chronic ethanol feeding to rats and the isolation and culture of Kupffer cells were performed as previously described^42^^.^ Briefly, rats were weight matched and then randomly assigned to pair-fed or ethanol-fed groups. Rats were allowed free access to the Lieber-DeCarli high-fat liquid diet (#710260, Dyets, Bethlehem, PA) for 2 days. Ethanol-fed rats were allowed free access to a liquid diet containing 17% of the calories (3.3% vol/vol) from ethanol for 2 days and then a liquid diet containing 35% of the calories (6.7% vol/vol) from ethanol for 4 weeks. Control rats were pair-fed a liquid diet in which maltose dextrin was substituted isocalorically for ethanol over the entire feeding period.

### Isolation and culture of Kupffer cells

Kupffer cells were isolated and cultured, as previously described^42^. After 18 hours in culture, cells were treated with or without 100 μg/ml small-specific-sized HA of average molecular weight of 7kD (HA7) or 35kD (HA35) (Lifecore Biomedical, LLC, Chaska, MN) for 5 h. Kupffer cells were then challenged with 10 ng/mL LPS (*Escherichia Coli* strain 0111:B4, Sigma-Aldrich) or PamCys3K, as indicated in the figure legends. For experiments using miRNA hairpin inhibitor or siRNA, Kupffer cells were nucleofected using the Amaxa mouse macrophage Nucleofector kit using the Y-001 program (Lonza, Cologne, Germany), as previously reported^43^. After nucleofection, cells retained sensitivity to LPS, ethanol and HA35; however, the extent of expression of TNFα was lower than in non-transfected cells (data not shown). Validated ON TARGET plus specific siRNAs SMART pool (rat CD44, cat no. L-089166-02-0005; rat TLR4, cat no. L-09819-02-0005; rat RHAMM, cat no. L-089927-02-0005 and rat TLR2, cat no. L-092211-02-0005) were purchased from Dharmacon, GE. Sequences of miRNAs primers and hairpin inhibitor are listed in Supplemental Table 2.

### Short-term ethanol feeding to mice

Ten to twelve week old female C57BL6/J mice were purchased from Jackson Laboratories (Bar Harbor, ME) and allowed free access to a Lieber-DeCarli liquid diet containing ethanol or a pair-fed control diet that isocalorically substituted maltose dextrin for ethanol^8^. Mice were housed two per micro-isolator cage and were maintained in a temperature regulated facility with a 12 hour light-dark cycle, with Nylabones provided for environmental enrichment. Mice were weight matched and randomly distributed to ethanol or control diets. They were then introduced to the control liquid diet for two days. Ethanol diets were initiated at 1% (v/v) for two days and then increased to 6% (v/v) or 32% of total calories for an additional two days. During the last three days of feeding, mice were gavaged with 15 mg/kg HA35 in sterile saline or an equivalent volume of saline (vehicle) at 1:30 pm. The morning after the last HA35 treatment, ethanol- and pair-fed mice were anesthetized and samples collected prior to euthanasia. The livers were perfused, excised and sectioned for FISH analysis.

### Human subjects

Patients with a confirmed diagnosis of alcoholic hepatitis were enrolled. The diagnosis of alcoholic hepatitis was performed by clinicians at the Cleveland Clinic and University Hospitals, Cleveland based on the patients’ medical history, physical examination, and laboratory data and according to the guidelines of the American College of Gastroenterology [https://gi.org/clinical-guidelines/]. Healthy control subjects were recruited from the Clinical Research Unit at the Cleveland Clinic. The study protocol was approved by the Institutional Review Board for the Protection of Human Subjects in Research at the Cleveland Clinic and University Hospitals, Cleveland. All methods were performed in accordance with the IRB’s guidelines and regulations and written informed consent was obtained from all subjects.

### Isolation and stimulation of human peripheral blood mononuclear cells (PBMCs)

Isolation of PBMCs from human blood was performed by density gradient centrifugation on Ficoll-Paque PLUS. Briefly, 1 mL of freshly collected Buffy Coat was mixed at a ratio of 1:1 (vol/vol) with PBS at room temperature and 2 mL of the mixture was layered to 10 mL Ficoll-Paque PLUS in 15 mL tubes. After centrifugation at 800 g for 30 min at 20 °C (no brake), buffy coat fractions were collected, pooled, resuspended in PBS and centrifuged at 800 g for 20 min at 20 °C. The pellets were resuspended in 10 mL of PBS and again centrifuged at 800 g for 20 min at 20 °C, the top 10 mL of platelet-rich solution was discarded and the rest was centrifuged at 800 g for 5 min at 20 °C. The harvested PBMCs were counted and cultured in RPMI-1640 supplemented with 0.02 M HEPES, 100 μM Penicillin-streptomycin and 10 vol% heat-inactivated FBS in 96-well plates in humidified atmosphere (5% CO_2_, 37 °C). After 18 h, cells were treated with fresh culture media with or without 100 μg/ml HA35 for 5 h and then challenged with 10 ng/ml LPS for another 60 min.

### RNA isolation and quantitative real-time PCR (qRT-PCR)

Total RNA was isolated using the Qiagen RNeasy mini kit, reverse transcribed and qRT-PCR amplification was performed. The relative amount of target mRNA was determined using the comparative threshold (Ct) method by normalizing target mRNA Ct values to those of 18 S.

### miRNA preparation and Next Generation Sequencing (NGS)

Small RNA was isolated using miRNeasy Mini Kit (Qiagen, Germantown, MD) and sequenced following the Small RNA Sample Preparation Protocol (Illumina, San Diego, USA). The library was prepared from 10 ng of total RNA per sample according to the manufacturers instructions (TruSeq, Small RNAKit, Illumina). Single-stranded cDNAs were created with SuperScriptII Reverse Transcriptase and double-stranded cDNAs generated by PCR using adapter specific primers. Purified libraries were quantified and qualified using the High Sensitivity DNA Kit on a 2100 Bioanalyzer (Agilent Technologies, Böblingen, Germany). Sequencing of the libraries was carried out at the Genomic Core Facility (Cleveland Clinic) utilizing HiSeq. 2000 (Illumina). After sequencing, the data were obtained in Illumina FASTQ format. The data were analyzed using NGS small RNA sequence analysis software (version 2.5.1) using the rat build rn4MiRNA program (see Saikia, *et al*., 2017 for data analysis pipeline). MicroRNA array data have been deposited in NCBI’s Gene Expression Omnibus (accession number GSE95403).

### Immunocytochemistry of Kupffer cells

Expression of cell surface receptors TLR2, TLR4, CD44, and RHAMM was detected by immunocytochemistry^44^. Briefly, after nucleofection with siRNA, Kupffer cells were plated on Lab-Tek chamber slides. After culture for 24 h, cells were fixed using 4% paraformaldehyde, washed with phosphate-buffered saline (PBS) and permeabilized with 0.1% Triton-X-100 for 10 minutes. Cells were then washed with PBS, quenched with glycine, blocked for 1 hour and incubated overnight with primary antibodies (see Supplemental Table 2 antibody sources) at 4 °C. Cells were then washed with PBS and incubated in the dark with secondary antibodies (Supplemental Table 2) for 1 hour at room temperature. Cells were again washed with PBS and mounted with a DAPI-containing mounting media. Images were acquired using a Leica-confocal microscope using 40X objectives.

### Western blot analysis

Primary cultures of Kupffer cells were treated with or without 100 μg/ml HA35 for 5 hrs and then challenged with LPS (10 ng/ml) for another 1 hr. Cells were then moved to ice, washed with 2 ml ice-cold PBS buffer and then lysed in RIPA buffer (25 mM Tris-HCl, pH 7.6, 150 mM NaCl, 1% NP-40, 0.1% SDS) containing 1 mM sodium orthovanadate, 10 mM NaF, 10 mM sodium pyrophosphate, 10 mM β-glycerophosphate, 1 mM PMSF, 10 µg/ml aprotinin and protease inhibitor. Lysates were then centrifuged at 16,000 × g for 15 min at 4 °C. For Western blot analysis, 10 µg of cellular protein was separated on 10% SDS-PAGE gels and then transferred to PVDF membranes for immunoblotting. Membranes were blocked in BSA or non-fat milk and probed with specific antibodies against Tollip and HSC70 as a loading control. Western blot analysis was performed using enhanced chemiluminescence for signal detection. Signal intensities were quantified by densitometry using Image J software (NIH). To confirm equal loading of protein, blots were probed with antibodies specific for HSC70.

### *In situ* hybridization of micro-RNA 291b in mouse liver using LNA probes

Making use of 5′, 3′ digoxigenin-tagged Locked nucleic acid (LNA) probe, obtained from Exiqon (Denmark), miR291b was visualized in paraffin-embedded livers from pair- or ethanol-fed mice^45^. Formalin-fixed paraffin-embedded liver sections were de-paraffinized, washed with DEPC-PBS and subjected to antigen retrieval using proteinase-K treatment (20 µg/ml) for 15 minutes at room temperature. Sections were then washed with DEPC-PBS and treated with 1-ethyl-3-(dimethylaminopropyl) carbodiimide-hydrochloride (EDC, Molecular Probes) for 90 minutes followed by sequential washing with 0.1 M Glycine/PBS for 10 minutes and DEPC-PBS, 3 times for 5 minutes each. Sections were then subjected to pre-hybridization for one hour at 40 °C followed by hybridization using 60 nM LNA-probe-containing hybridization buffer (50% formamide, 5x saline sodium citrate, 0.1% Tween-20, adjusted to pH 6.0 with 9.2 mM citric acid, 500 ug/ml yeast tRNA) for one hour at 45 °C in a humidified chamber. At the end of the hybridization, sections were sequentially treated with ice-cold SSC buffer (containing sodium chloride and citrate buffer), 3% H_2_O_2_ for 10 minutes, DEPC-PBS and *in situ* hybridization-blocking buffer from Roche (Mannheim, Germany Cat. No.11-096-176-001) for 1 hour. Sections were then exposed to anti-digoxigenin-HRP labeled antibody for 1 hour at room temperature. After the antibody incubation, sections were washed with TN-buffer followed by incubation with Tyramide signal amplification (TSA) reagent for 10 minutes in dark, washed three time with TN buffer and mounted using DAPI-containing mounting reagent. Images were acquired using 40X objective.

### Statistical analysis

Values shown in all figures represent means + SEM. Data was analyzed by Analysis of Variance using a general linear model and least square means test for comparisons between groups (SAS, Carey, IN). Normal distribution of the data was assessed using the Shapiro-Wilk test and data were log-transformed, if necessary, to obtain a normal distribution. Statistical difference between groups was determined at p < 0.05. Different alphabetical superscripts denote significant difference between groups.

## Electronic supplementary material

Supplemental Information
