# Supplementary material for: Hyaluronic acid 35 normalizes TLR4 signaling in Kupffer cells from ethanol-fed rats via regulation of microRNA291b and its target Tollip
Source: Sci Rep. 2017 Nov 15;7:15671. doi: 10.1038/s41598-017-15760-4 (PMC5688113; doi:10.1038/s41598-017-15760-4)
Supplement: Supplementary file 1 — Supplemental Information [file 41598_2017_15760_MOESM1_ESM.pdf]

Supplemental Information for:

Hyaluronic acid 35 normalizes TLR4 signalling in Kupffer cells from ethanol-fed rats via regulation of microRNA291b and its target Tollip

Paramananda Saikia [1,3], Sanjoy Roychowdhury[1,3], Damien Bellos[1,3], Katherine A. Pollard[1], Megan R. McMullen[1], Rebecca L. McCullough[1], Arthur J. McCullough[1,2] Pierre Gholam[4], Carol de la Motte[1,3] and Laura E. Nagy[1,2,3]

Center for Liver Disease Research, Departments of [1]Pathobiology and [2]Gastroenterology, Cleveland Clinic, Cleveland OH

[3]Department of Molecular Medicine, Case Western Reserve University, Cleveland, OH

[4]Department of Gastroenterology and Hepatology, University Hospital, Cleveland, OH

Supplemental Figure legends

Supplemental Figure 1: siRNA knockdown of HA TLR4 in Kupffer cells from pair- and ethanol-fed rats. Wistar rats were allowed free access to a Lieber-DeCarli ethanol diet or pair-fed control diet for 4 weeks. Kupffer cells were isolated and nucleofected with 25 nM of scrambled siRNA or siRNA targeted to TLR4. 18h post-nucleofection, Kupffer cells were treated with or without 100 µg/ml HA35 for 5h and then challenged with 10 ng/ml LPS for 1hr and TNFα mRNA measured by qRT-PCR and normalized to 18S rRNA. Values represent means ± SEM, n=4, *p<0.05 compared to pair-fed within a treatment group.

Supplemental Figure 2: Full size gel images for Figure 4 A/B: Regulation of Tollip expression by ethanol, HA35 and miR291b in primary cultures of Kupffer cells. (A) Kupffer cells isolated from ethanol- and pair-fed rats were cultured overnight and then treated with 100 µg/ml HA35 for 5 h and then challenged with 10 ng/ml LPS for an additional 60 min. **(B)** Kupffer cells isolated from ethanol- and pair-fed rats were nucleofected with either control hairpin or miR291b hairpin inhibitor 18 h post-nucleofection, Kupffer cells were treated or not with 100 µg/ml HA35 for 5 h and then challenged with 10 ng/ml LPS for an additional 1hr. **(A/B)** Kupffer cells were lysed and Tollip expression measured by Western blot. HSC70 was used as a loading control.

Supplemental Figure 1

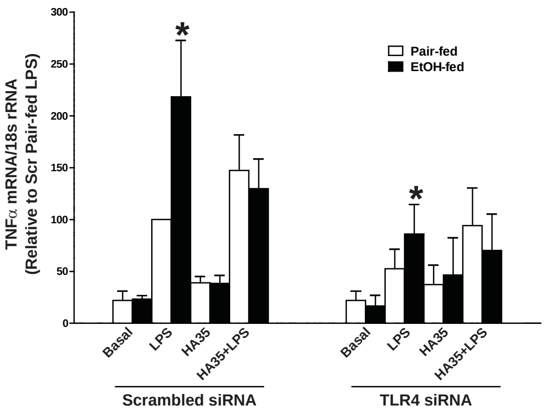

Figure 5A

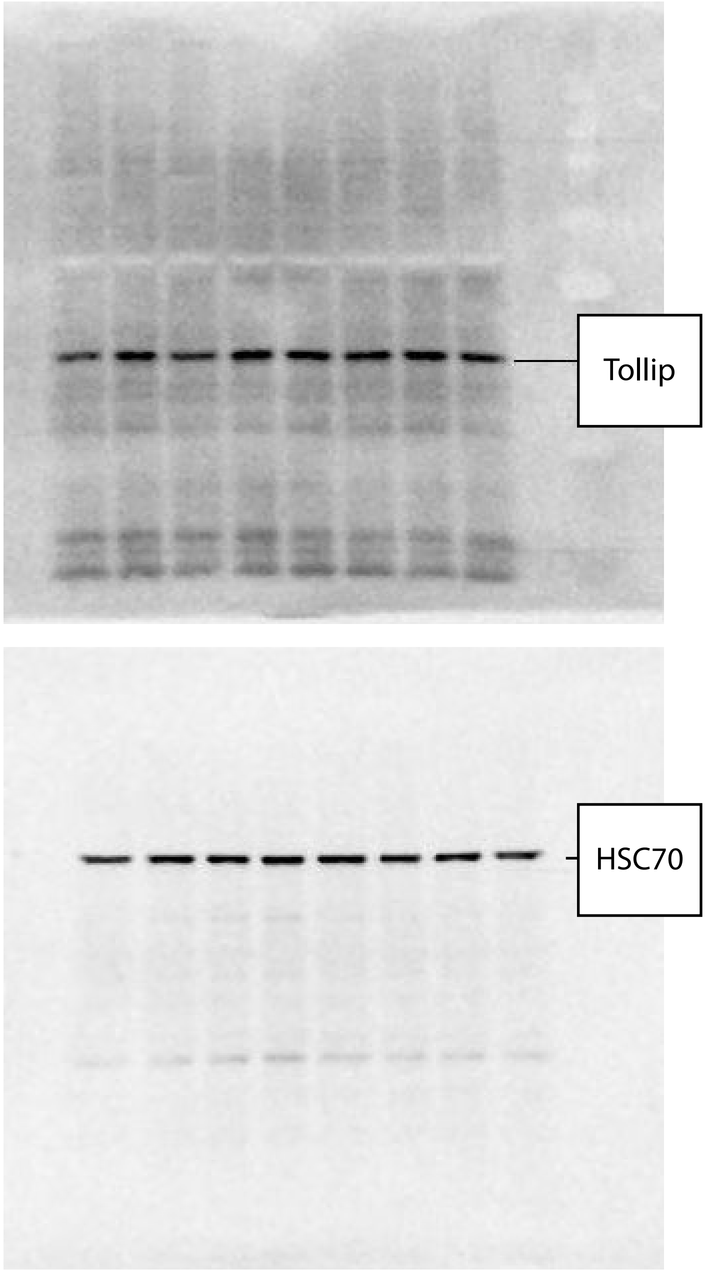

Figure 5B

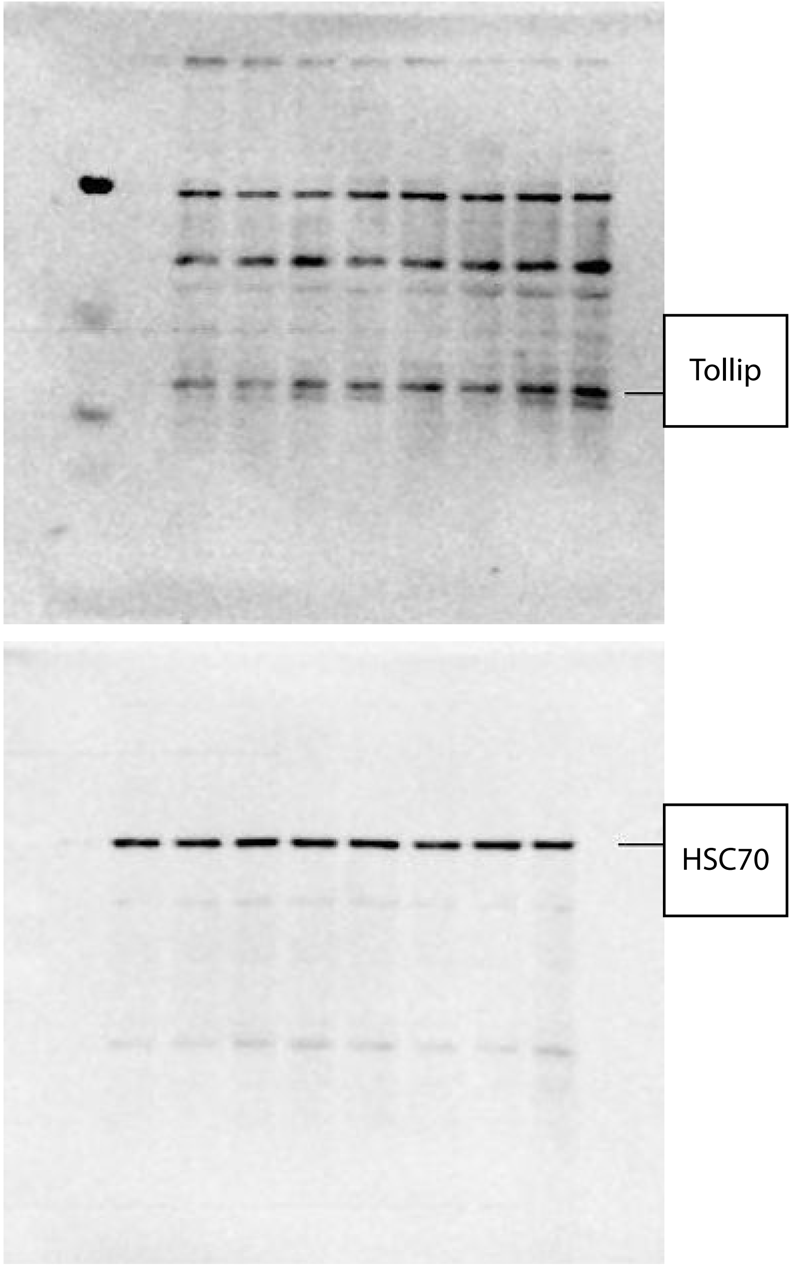

Supplemental Table 1: *In silico* analysis using MiRDB (www.mirdb.org): Predicted miR291b targeted genes and their target scores.

Target Score	miRNA Name	Gene Symbol	Gene Description
100	rno-miR-291b	Zfyve9	zinc finger, FYVE domain containing 9
100	rno-miR-291b	Kdm2a	lysine (K)-specific demethylase 2A
100	rno-miR-291b	Lpgat1	lysophosphatidylglycerol acyltransferase 1
99	rno-miR-291b	Arhgap12	Rho GTPase activating protein 12
99	rno-miR-291b	Cmpk1	cytidine monophosphate (UMP-CMP) kinase 1
99	rno-miR-291b	Cpeb1	cytoplasmic polyadenylation element binding protein 1
99	rno-miR-291b	R3hdm1	R3H domain containing 1
99	rno-miR-291b	Fam73b	family with sequence similarity 73, member B
99	rno-miR-291b	Lclat1	lysocardiolipin acyltransferase 1
99	rno-miR-291b	Arid4b	AT rich interactive domain 4B (Rbp1 like)
99	rno-miR-291b	Dnajb9	DnaJ (Hsp40) homolog, subfamily B, member 9
98	rno-miR-291b	Clcn4	chloride channel, voltage-sensitive 4
98	rno-miR-291b	Rasd1	RAS, dexamethasone-induced 1
98	rno-miR-291b	Mtmr4	myotubularin related protein 4
98	rno-miR-291b	Pdgfra	platelet derived growth factor receptor, alpha polypeptide
98	rno-miR-291b	Mier3	mesoderm induction early response 1, family member 3
98	rno-miR-291b	Naa30	N(alpha)-acetyltransferase 30, NatC catalytic subunit
98	rno-miR-291b	Zfyve20	zinc finger, FYVE domain containing 20
98	rno-miR-291b	Fcho2	FCH domain only 2
98	rno-miR-291b	Wdr20	WD repeat domain 20
98	rno-miR-291b	Ankib1	ankyrin repeat and IBR domain containing 1
97	rno-miR-291b	Lmx1a	LIM homeobox transcription factor 1 alpha
97	rno-miR-291b	Slain1	SLAIN motif family, member 1
97	rno-miR-291b	Usp32	ubiquitin specific peptidase 32
97	rno-miR-291b	Stx6	syntaxin 6
97	rno-miR-291b	Fmr1	fragile X mental retardation 1
97	rno-miR-291b	Pxk	PX domain containing serine/threonine kinase
97	rno-miR-291b	Bmpr2	bone morphogenetic protein receptor, type II (serine/threonine kinase)
97	rno-miR-291b	Ndel1	nudE neurodevelopment protein 1-like 1
97	rno-miR-291b	Acsl4	acyl-CoA synthetase long-chain family member 4
97	rno-miR-291b	Psd3	pleckstrin and Sec7 domain containing 3
97	rno-miR-291b	Ccdc88a	coiled coil domain containing 88A
96	rno-miR-291b	Hivep2	human immunodeficiency virus type I enhancer binding protein 2
96	rno-miR-291b	Tsg101	tumor susceptibility 101
96	rno-miR-291b	Mapk8	mitogen-activated protein kinase 8
96	rno-miR-291b	Rfx5	regulatory factor X, 5 (influences HLA class II expression)
96	rno-miR-291b	Cdk2	cyclin dependent kinase 2
96	rno-miR-291b	Rnf216	ring finger protein 216
95	rno-miR-291b	Laptm4a	lysosomal protein transmembrane 4 alpha
95	rno-miR-291b	Chd5	chromodomain helicase DNA binding protein 5
95	rno-miR-291b	Fibin	fin bud initiation factor homolog (zebrafish)
95	rno-miR-291b	Nkiras1	NFKB inhibitor interacting Ras-like 1
95	rno-miR-291b	Arhgap1	Rho GTPase activating protein 1
95	rno-miR-291b	Cmpk2	cytidine monophosphate (UMP-CMP) kinase 2, mitochondrial
95	rno-miR-291b	Jakmip1	janus kinase and microtubule interacting protein 1
95	rno-miR-291b	Slc40a1	solute carrier family 40 (iron-regulated transporter), member 1
95	rno-miR-291b	Sall3	spalt-like transcription factor 3
95	rno-miR-291b	Map3k12	mitogen activated protein kinase kinase kinase 12
95	rno-miR-291b	Atg16l1	autophagy related 16-like 1 (S. cerevisiae)
94	rno-miR-291b	Synm	synemin, intermediate filament protein
94	rno-miR-291b	Btf3l4	basic transcription factor 3-like 4
94	rno-miR-291b	Socs6	suppressor of cytokine signaling 6
94	rno-miR-291b	Map3k8	mitogen-activated protein kinase kinase kinase 8
94	rno-miR-291b	Ago1	argonaute RISC catalytic component 1
94	rno-miR-291b	Btg3	BTG family, member 3

Supplemental Table 1: *In silico* analysis using MiRDB (www.mirdb.org): Predicted miR291b targeted genes and their target scores.

94	rno-miR-291b	Psd	pleckstrin and Sec7 domain containing
94	rno-miR-291b	Elk4	ELK4, ETS-domain protein (SRF accessory protein 1)
94	rno-miR-291b	Fbxl5	F-box and leucine-rich repeat protein 5
94	rno-miR-291b	Slc24a2	solute carrier family 24 (sodium/potassium/calcium exchanger), member 2
94	rno-miR-291b	Epha4	Eph receptor A4
94	rno-miR-291b	Sos1	Son of sevenless homolog 1 (Drosophila)
94	rno-miR-291b	Fbxo48	F-box protein 48
93	rno-miR-291b	Pdik1l	PDLIM1 interacting kinase 1 like
93	rno-miR-291b	Kbtbd2	kelch repeat and BTB (POZ) domain containing 2
93	rno-miR-291b	Rtn1	reticulon 1
93	rno-miR-291b	Cul3	cullin 3
93	rno-miR-291b	Zfhx4	zinc finger homeobox 4
92	rno-miR-291b	Ube3a	ubiquitin protein ligase E3A
92	rno-miR-291b	Rab10	RAB10, member RAS oncogene family
92	rno-miR-291b	Slc25a10	solute carrier family 25 (mitochondrial carrier; dicarboxylate transporter), member 10
92	rno-miR-291b	Crebzf	CREB/ATF bZIP transcription factor
92	rno-miR-291b	Bicd2	bicaudal D homolog 2 (Drosophila)
92	rno-miR-291b	Arhgap24	Rho GTPase activating protein 24
92	rno-miR-291b	Suv420h1	suppressor of variegation 4-20 homolog 1 (Drosophila)
92	rno-miR-291b	Polq	polymerase (DNA directed), theta
92	rno-miR-291b	Prkaa1	protein kinase, AMP-activated, alpha 1 catalytic subunit
91	rno-miR-291b	Tesk2	testis-specific kinase 2
91	rno-miR-291b	Lzic	leucine zipper and CTNNBIP1 domain containing
91	rno-miR-291b	Blcap	bladder cancer associated protein
91	rno-miR-291b	Map3k2	mitogen activated protein kinase kinase kinase 2
91	rno-miR-291b	Emx2	empty spiracles homeobox 2
91	rno-miR-291b	LOC100363065	zinc finger protein 11
91	rno-miR-291b	Gca	grancalcin
91	rno-miR-291b	Ddhd2	DDHD domain containing 2
90	rno-miR-291b	LOC678772	similar to O-acetyltransferase
90	rno-miR-291b	Trim3	tripartite motif-containing 3
90	rno-miR-291b	Smoc2	SPARC related modular calcium binding 2
90	rno-miR-291b	Stk17b	serine/threonine kinase 17b
90	rno-miR-291b	Rasl11b	RAS-like family 11 member B
90	rno-miR-291b	Pkia	protein kinase (cAMP-dependent, catalytic) inhibitor alpha
90	rno-miR-291b	LOC102555051	zinc finger protein 709-like
90	rno-miR-291b	Myocd	myocardin
89	rno-miR-291b	Cnnm3	cyclin M3
89	rno-miR-291b	Pex19	peroxisomal biogenesis factor 19
89	rno-miR-291b	Klhl20	kelch-like family member 20
89	rno-miR-291b	Gabrr1	gamma-aminobutyric acid (GABA) A receptor, rho 1
89	rno-miR-291b	Epha5	EPH receptor A5
89	rno-miR-291b	Atrn	attractin
89	rno-miR-291b	Tnfrsf21	tumor necrosis factor receptor superfamily, member 21
88	rno-miR-291b	Rb1	retinoblastoma 1
88	rno-miR-291b	Mapre1	microtubule-associated protein, RP/EB family, member 1
88	rno-miR-291b	Frmd6	FERM domain containing 6
88	rno-miR-291b	Sumf1	sulfatase modifying factor 1
88	rno-miR-291b	Tp63	tumor protein p63
88	rno-miR-291b	Cnot7	CCR4-NOT transcription complex, subunit 7
88	rno-miR-291b	Atxn1l	ataxin 1-like
88	rno-miR-291b	Nckap5	NCK-associated protein 5
88	rno-miR-291b	Eea1	early endosome antigen 1
88	rno-miR-291b	Cnot6l	CCR4-NOT transcription complex, subunit 6-like
87	rno-miR-291b	Mex3d	mex-3 RNA binding family member D
87	rno-miR-291b	Usp3	ubiquitin specific peptidase 3
87	rno-miR-291b	Tppp	tubulin polymerization promoting protein

Supplemental Table 1: *In silico* analysis using MiRDB (www.mirdb.org): Predicted miR291b targeted genes and their target scores.

87	rno-miR-291b	2-Sep	septin 2
87	rno-miR-291b	Pcsk5	proprotein convertase subtilisin/kexin type 5
87	rno-miR-291b	Kif23	kinesin family member 23
87	rno-miR-291b	Wdfy3	WD repeat and FYVE domain containing 3
87	rno-miR-291b	Pthlh	parathyroid hormone-like hormone
87	rno-miR-291b	Rapgef4	Rap guanine nucleotide exchange factor (GEF) 4
87	rno-miR-291b	Arhgap6	Rho GTPase activating protein 6
86	rno-miR-291b	Vangl1	VANGL planar cell polarity protein 1
86	rno-miR-291b	Skida1	SKI/DACH domain containing 1
86	rno-miR-291b	Wee1	WEE1 G2 checkpoint kinase
86	rno-miR-291b	Ddx5	DEAD (Asp-Glu-Ala-Asp) box helicase 5
86	rno-miR-291b	Setd5	SET domain containing 5
86	rno-miR-291b	Klhl28	kelch-like family member 28
86	rno-miR-291b	Bag5	BCL2-associated athanogene 5
86	rno-miR-291b	Hif1a	hypoxia-inducible factor 1, alpha subunit (basic helix-loop-helix transcription factor)
86	rno-miR-291b	Tox3	TOX high mobility group box family member 3
86	rno-miR-291b	Csgalnact1	chondroitin sulfate N-acetylgalactosaminyltransferase 1
86	rno-miR-291b	Tnks1bp1	tankyrase 1 binding protein 1
85	rno-miR-291b	Rock2	Rho-associated coiled-coil containing protein kinase 2
85	rno-miR-291b	Tspyl2	TSPY-like 2
85	rno-miR-291b	Nabp1	nucleic acid binding protein 1
85	rno-miR-291b	Abhd3	abhydrolase domain containing 3
85	rno-miR-291b	Sfmbt1	Scm-like with four mbt domains 1
85	rno-miR-291b	Topors	topoisomerase I binding, arginine/serine-rich, E3 ubiquitin protein ligase
85	rno-miR-291b	Reps2	RALBP1 associated Eps domain containing 2
85	rno-miR-291b	Socs7	suppressor of cytokine signaling 7
85	rno-miR-291b	Snrk	SNF related kinase
85	rno-miR-291b	Ano3	anoctamin 3
84	rno-miR-291b	Acer2	alkaline ceramidase 2
84	rno-miR-291b	Npas2	neuronal PAS domain protein 2
84	rno-miR-291b	Prr14l	proline rich 14-like
84	rno-miR-291b	Nagk	N-acetylglucosamine kinase
84	rno-miR-291b	Naa50	N(alpha)-acetyltransferase 50, NatE catalytic subunit
84	rno-miR-291b	Mfsd6	major facilitator superfamily domain containing 6
84	rno-miR-291b	Prrt2	proline-rich transmembrane protein 2
84	rno-miR-291b	Apcdd1	adenomatosis polyposis coli down-regulated 1
83	rno-miR-291b	Sybu	syntabulin (syntaxin-interacting)
83	rno-miR-291b	LOC259246	alpha-2u globulin PGCL1
83	rno-miR-291b	Pkd2	polycystic kidney disease 2 (autosomal dominant)
83	rno-miR-291b	Panx2	pannexin 2
83	rno-miR-291b	Atp2c1	ATPase, Ca++ transporting, type 2C, member 1
83	rno-miR-291b	Btbd10	BTB (POZ) domain containing 10
83	rno-miR-291b	Efr3a	EFR3 homolog A (S. cerevisiae)
83	rno-miR-291b	Gcc2	GRIP and coiled-coil domain containing 2
83	rno-miR-291b	Hbp1	HMG-box transcription factor 1
83	rno-miR-291b	Itga4	integrin, alpha 4
83	rno-miR-291b	Btbd7	BTB (POZ) domain containing 7
82	rno-miR-291b	Hnrnpul1	heterogeneous nuclear ribonucleoprotein U-like 1
82	rno-miR-291b	Nek9	NIMA-related kinase 9
82	rno-miR-291b	Uxs1	UDP-glucuronate decarboxylase 1
82	rno-miR-291b	Nup35	nucleoporin 35
82	rno-miR-291b	Elk3	ELK3, member of ETS oncogene family
82	rno-miR-291b	Atp2b2	ATPase, Ca++ transporting, plasma membrane 2
82	rno-miR-291b	Fam169a	family with sequence similarity 169, member A
81	rno-miR-291b	Ccdc8	coiled-coil domain containing 8
81	rno-miR-291b	Ankrd12	ankyrin repeat domain 12
81	rno-miR-291b	Sema4b	sema domain, immunoglobulin domain (Ig), transmembrane domain (TM) and short cytoplasmic domain, (semaphorin) 4B

Supplemental Table 1: *In silico* analysis using MiRDB (www.mirdb.org): Predicted miR291b targeted genes and their target scores.

81	rno-miR-291b	Sh3bp2	SH3-domain binding protein 2
81	rno-miR-291b	Slc19a2	solute carrier family 19 (thiamine transporter), member 2
81	rno-miR-291b	Hs3st5	heparan sulfate (glucosamine) 3-O-sulfotransferase 5
81	rno-miR-291b	Rad51b	RAD51 paralog B
80	rno-miR-291b	Actl6a	actin-like 6A
80	rno-miR-291b	Phtf2	putative homeodomain transcription factor 2
80	rno-miR-291b	Rorb	RAR-related orphan receptor B
80	rno-miR-291b	Hif1an	hypoxia-inducible factor 1, alpha subunit inhibitor
80	rno-miR-291b	Rbbp6	retinoblastoma binding protein 6
80	rno-miR-291b	Myrf	myelin regulatory factor
80	rno-miR-291b	Papola	poly (A) polymerase alpha
79	rno-miR-291b	Srsf2	serine/arginine-rich splicing factor 2
79	rno-miR-291b	Mastl	microtubule associated serine/threonine kinase-like
79	rno-miR-291b	Fam78a	family with sequence similarity 78, member A
79	rno-miR-291b	Hmgb3	high mobility group box 3
79	rno-miR-291b	Nell2	NEL-like 2 (chicken)
79	rno-miR-291b	Maml1	mastermind like 1 (Drosophila)
79	rno-miR-291b	Gpr158	G protein-coupled receptor 158
79	rno-miR-291b	Zbtb4	zinc finger and BTB domain containing 4
79	rno-miR-291b	Fam45a	family with sequence similarity 45, member A
79	rno-miR-291b	Map1b	microtubule-associated protein 1B
79	rno-miR-291b	Dpysl2	dihydropyrimidinase-like 2
79	rno-miR-291b	Tollip	toll interacting protein
79	rno-miR-291b	Golga1	golgin A1
78	rno-miR-291b	LOC102549010	BEN domain-containing protein 2-like
78	rno-miR-291b	Zfpm2	zinc finger protein, multitype 2
78	rno-miR-291b	Scamp2	secretory carrier membrane protein 2
78	rno-miR-291b	Uchl5	ubiquitin carboxyl-terminal hydrolase L5
78	rno-miR-291b	Hoxa3	homeobox A3
78	rno-miR-291b	Sall1	spalt-like transcription factor 1
78	rno-miR-291b	Has2	hyaluronan synthase 2
78	rno-miR-291b	Styxl2	serine/threonine/tyrosine interacting protein-like2
77	rno-miR-291b	Slc15a1	solute carrier family 15 (oligopeptide transporter), member 1
77	rno-miR-291b	Ttc8	tetratricopeptide repeat domain 8
77	rno-miR-291b	Trappc2	trafficking protein particle complex 2
77	rno-miR-291b	Wasf1	WAS protein family, member 1
77	rno-miR-291b	Itpr1	inositol 1,4,5-trisphosphate receptor, type 1
77	rno-miR-291b	Camsap1	calmodulin regulated spectrin-associated protein 1
77	rno-miR-291b	Mcm7	minichromosome maintenance complex component 7
77	rno-miR-291b	Nus1	nuclear undecaprenyl pyrophosphate synthase 1 homolog (S. cerevisiae)
77	rno-miR-291b	Ankrd29	ankyrin repeat domain 29
76	rno-miR-291b	Tex2	testis expressed 2
76	rno-miR-291b	Tes	testis derived transcript
76	rno-miR-291b	Pgp	phosphoglycolate phosphatase
76	rno-miR-291b	Itgb4	integrin, beta 4
76	rno-miR-291b	Ndufs1	NADH dehydrogenase (ubiquinone) Fe-S protein 1
76	rno-miR-291b	Cep57	centrosomal protein 57
76	rno-miR-291b	Kcnd2	potassium voltage-gated channel, Shal-related subfamily, member 2
76	rno-miR-291b	Mfap3l	microfibrillar-associated protein 3-like
76	rno-miR-291b	Lurap1	leucine rich adaptor protein 1
76	rno-miR-291b	Shank2	SH3 and multiple ankyrin repeat domains 2
76	rno-miR-291b	Cpeb3	cytoplasmic polyadenylation element binding protein 3
76	rno-miR-291b	LOC683071	similar to La-related protein 4 (La ribonucleoprotein domain family member 4)
75	rno-miR-291b	Bahd1	bromo adjacent homology domain containing 1
75	rno-miR-291b	RGD1304884	similar to RIKEN cDNA 6430548M08
75	rno-miR-291b	Fzd6	frizzled family receptor 6
75	rno-miR-291b	Ispd	isoprenoid synthase domain containing

Supplemental Table 1: *In silico* analysis using MiRDB (www.mirdb.org): Predicted miR291b targeted genes and their target scores.

75	rno-miR-291b	Lrrc4c	leucine rich repeat containing 4C
75	rno-miR-291b	Vldlr	very low density lipoprotein receptor
75	rno-miR-291b	Dync1li2	dynein, cytoplasmic 1 light intermediate chain 2
74	rno-miR-291b	Lima1	LIM domain and actin binding 1
74	rno-miR-291b	St6galnac3	ST6 (alpha-N-acetyl-neuraminyl-2,3-beta-galactosyl-1,3)-N-acetylgalactosaminide alpha-2,6-sialyltransferase 3
74	rno-miR-291b	Pdcd1lg2	programmed cell death 1 ligand 2
74	rno-miR-291b	Dip2a	DIP2 disco-interacting protein 2 homolog A (Drosophila)
74	rno-miR-291b	Frk	fyn-related kinase
74	rno-miR-291b	Klf9	Kruppel-like factor 9
73	rno-miR-291b	Med14	mediator complex subunit 14
73	rno-miR-291b	Dgkb	diacylglycerol kinase, beta
73	rno-miR-291b	Larp4b	La ribonucleoprotein domain family, member 4B
73	rno-miR-291b	Snx5	sorting nexin 5
73	rno-miR-291b	Ogt	O-linked N-acetylglucosamine (GlcNAc) transferase
73	rno-miR-291b	Bmp2k	BMP-2 inducible kinase
73	rno-miR-291b	Tmem196	transmembrane protein 196
73	rno-miR-291b	Scn1a	sodium channel, voltage-gated, type I, alpha
73	rno-miR-291b	Abi1	abl-interactor 1
73	rno-miR-291b	Mtbp	Mdm2, transformed 3T3 cell double minute 2, p53 binding protein (mouse) binding protein
73	rno-miR-291b	Mapk4	mitogen-activated protein kinase 4
73	rno-miR-291b	Rnf181	ring finger protein 181
73	rno-miR-291b	Tmem50b	transmembrane protein 50B
73	rno-miR-291b	Limk1	LIM domain kinase 1
73	rno-miR-291b	Pard6b	par-6 family cell polarity regulator beta
72	rno-miR-291b	Tlx1	T-cell leukemia, homeobox 1
72	rno-miR-291b	Tfcp2l1	transcription factor CP2-like 1
72	rno-miR-291b	Hars2	histidyl-tRNA synthetase 2, mitochondrial
72	rno-miR-291b	Slc18a2	solute carrier family 18 (vesicular monoamine transporter), member 2
72	rno-miR-291b	Sgip1	SH3-domain GRB2-like (endophilin) interacting protein 1
72	rno-miR-291b	Fgf9	fibroblast growth factor 9
72	rno-miR-291b	Cep97	centrosomal protein 97
71	rno-miR-291b	Jam2	junctional adhesion molecule 2
71	rno-miR-291b	Gml	glycosylphosphatidylinositol anchored molecule like
71	rno-miR-291b	Ptpn4	protein tyrosine phosphatase, non-receptor type 4
71	rno-miR-291b	Pknox1	PBX/knotted 1 homeobox 1
71	rno-miR-291b	Zfp512b	zinc finger protein 512B
71	rno-miR-291b	Arhgap35	Rho GTPase activating protein 35
71	rno-miR-291b	Casp8	caspase 8
71	rno-miR-291b	Hook3	hook microtubule-tethering protein 3
70	rno-miR-291b	Pfn2	profilin 2
70	rno-miR-291b	RGD1305254	similar to transmembrane protein 2
70	rno-miR-291b	Sult1c3	sulfotransferase family, cytosolic, 1C, member 3
70	rno-miR-291b	Yaf2	YY1 associated factor 2
70	rno-miR-291b	Birc6	baculoviral IAP repeat-containing 6
70	rno-miR-291b	Fam63b	family with sequence similarity 63, member B
69	rno-miR-291b	Nfat5	nuclear factor of activated T-cells 5, tonicity-responsive
69	rno-miR-291b	Ythdf2	YTH domain family, member 2
69	rno-miR-291b	Mlc1	megalencephalic leukoencephalopathy with subcortical cysts 1
69	rno-miR-291b	Slc41a1	solute carrier family 41 (magnesium transporter), member 1
69	rno-miR-291b	Fam196b	family with sequence similarity 196, member B
69	rno-miR-291b	Cox7a2l	cytochrome c oxidase subunit VIIa polypeptide 2 like
69	rno-miR-291b	Srgap2	SLIT-ROBO Rho GTPase activating protein 2
69	rno-miR-291b	Usp31	ubiquitin specific peptidase 31
69	rno-miR-291b	Xrn1	5'-3' exoribonuclease 1
69	rno-miR-291b	LOC100910235	sulfotransferase 1C1-like

Supplemental Table 1: *In silico* analysis using MiRDB (www.mirdb.org): Predicted miR291b targeted genes and their target scores.

69	rno-miR-291b	Pou2af1	POU class 2 associating factor 1
69	rno-miR-291b	Hey2	hes-related family bHLH transcription factor with YRPW motif 2
69	rno-miR-291b	Fam160b2	family with sequence similarity 160, member B2
68	rno-miR-291b	Vsig4	V-set and immunoglobulin domain containing 4
68	rno-miR-291b	Fsd1l	fibronectin type III and SPRY domain containing 1-like
67	rno-miR-291b	U2surp	U2 snRNP-associated SURP domain containing
67	rno-miR-291b	RGD1311558	similar to 4930506M07Rik protein
66	rno-miR-291b	Arid4a	AT rich interactive domain 4A (Rbp1 like)
66	rno-miR-291b	Rab5b	RAB5B, member RAS oncogene family
66	rno-miR-291b	Filip1l	filamin A interacting protein 1-like
66	rno-miR-291b	Dcaf8	DDB1 and CUL4 associated factor 8
66	rno-miR-291b	LOC102555532	uncharacterized LOC102555532
66	rno-miR-291b	Fastk	Fas-activated serine/threonine kinase
66	rno-miR-291b	Lrrtm2	leucine rich repeat transmembrane neuronal 2
66	rno-miR-291b	Thra	thyroid hormone receptor alpha
66	rno-miR-291b	Heg1	heart development protein with EGF-like domains 1
65	rno-miR-291b	Cep120	centrosomal protein 120
65	rno-miR-291b	Caps2	calcyphosine 2
65	rno-miR-291b	Ssh2	slingshot protein phosphatase 2
65	rno-miR-291b	Dnajc27	DnaJ (Hsp40) homolog, subfamily C, member 27
65	rno-miR-291b	Dpyd	dihydropyrimidine dehydrogenase
65	rno-miR-291b	Chmp4c	charged multivesicular body protein 4C
64	rno-miR-291b	Ppp1r3b	protein phosphatase 1, regulatory subunit 3B
64	rno-miR-291b	Leprot	leptin receptor overlapping transcript
64	rno-miR-291b	Ppp1r15b	protein phosphatase 1, regulatory subunit 15B
64	rno-miR-291b	Nr1d1	nuclear receptor subfamily 1, group D, member 1
64	rno-miR-291b	Sacs	spastic ataxia of Charlevoix-Saguenay (sacsin)
64	rno-miR-291b	Kif5a	kinesin family member 5A
64	rno-miR-291b	Pou4f3	POU class 4 homeobox 3
63	rno-miR-291b	Nbl1	neuroblastoma 1, DAN family BMP antagonist
63	rno-miR-291b	Heatr5a	HEAT repeat containing 5A
63	rno-miR-291b	Fam175b	family with sequence similarity 175, member B
63	rno-miR-291b	Megf9	multiple EGF-like-domains 9
63	rno-miR-291b	Ampd3	adenosine monophosphate deaminase 3
63	rno-miR-291b	Pcdhac2	protocadherin alpha subfamily C, 2
63	rno-miR-291b	RGD1562952	similar to Erbb2 interacting protein isoform 2
63	rno-miR-291b	Nhlh2	nescient helix loop helix 2
63	rno-miR-291b	Scoc	short coiled-coil protein
63	rno-miR-291b	Ppp6r1	protein phosphatase 6, regulatory subunit 1
63	rno-miR-291b	Zfp91	zinc finger protein 91
63	rno-miR-291b	Rgma	repulsive guidance molecule family member A
63	rno-miR-291b	Ms4a6a	membrane-spanning 4-domains, subfamily A, member 6A
63	rno-miR-291b	LOC100365043	histone cluster 1, H2bd-like
63	rno-miR-291b	Grb10	growth factor receptor bound protein 10
62	rno-miR-291b	Atf6	activating transcription factor 6
62	rno-miR-291b	Tspan9	tetraspanin 9
62	rno-miR-291b	Arhgap28	Rho GTPase activating protein 28
62	rno-miR-291b	Gramd1a	GRAM domain containing 1A
62	rno-miR-291b	Heca	headcase homolog (Drosophila)
62	rno-miR-291b	Lsm14b	LSM14B, SCD6 homolog B (S. cerevisiae)
62	rno-miR-291b	Arx	aristaless related homeobox
62	rno-miR-291b	Aldh1b1	aldehyde dehydrogenase 1 family, member B1
62	rno-miR-291b	Pitpna	phosphatidylinositol transfer protein, alpha
62	rno-miR-291b	Ezh1	enhancer of zeste homolog 1 (Drosophila)
62	rno-miR-291b	Atp1b4	ATPase, (Na+)/K+ transporting, beta 4 polypeptide
62	rno-miR-291b	Isoc1	isochorismatase domain containing 1
61	rno-miR-291b	Cstf2t	cleavage stimulation factor, 3' pre-RNA, subunit 2, 64kDa, tau variant
61	rno-miR-291b	Abhd12	abhydrolase domain containing 12
61	rno-miR-291b	Ube2g2	ubiquitin-conjugating enzyme E2G 2

61	rno-miR-291b	Eif4g2	eukaryotic translation initiation factor 4, gamma 2
61	rno-miR-291b	Arl6ip1	ADP-ribosylation factor-like 6 interacting protein 1
61	rno-miR-291b	Asf1a	anti-silencing function 1A histone chaperone
61	rno-miR-291b	Gon4l	gon-4-like (C. elegans)
61	rno-miR-291b	Cyyr1	cysteine/tyrosine-rich 1
60	rno-miR-291b	Fgfr1op2	FGFR1 oncogene partner 2
60	rno-miR-291b	Zfp367	zinc finger protein 367
60	rno-miR-291b	Sox4	SRY (sex determining region Y)-box 4
60	rno-miR-291b	Zfp167	zinc finger protein 167
60	rno-miR-291b	Cd69	Cd69 molecule
60	rno-miR-291b	N4bp1	Nedd4 binding protein 1
60	rno-miR-291b	LOC100912163	AT-rich interactive domain-containing protein 4B-like

Supplemental Table 2: Commercial sources and sequences for antibodies and primers

Antibody sources

**Primary Antibody against:**	**Company/catalog number**
rabbit anti-TLR4	Santa Cruz,sc-10741
goat anti-TLR2	Santa Cruz sc-16237
mouse anti-CD44	SIGMA, C7923
goat anti-RHAMM	Santa Cruz, sc-16170
mouse anti-HSC70	Santa Cruz sc-7298
mouse anti-Tollip	R&D System, MAB4678
**Secondary Antibody:**	
donkey anti-rabbit IgG 488	Molecular Probes, A21206
rabbit anti-goat IgG 568	Molecular Probes, A11078
goat anti-mouse IgG 594	Molecular Probes, A11032
goat anti-mouse	Santa Cruz sc-2005

Primer sequences (mRNA)

**Gene of interest**	**Forward**	**Reverse**
	**Rat**	
TNFα	CAA GGA GGA GAA GTT CCC AA	CTC TGC TTG GTG GTT TGC TA
18s	CTG AGA AAC GGC TAC CAC ATC	CAC CAC CAC CCA CGG AAT CG
	**Human**	
TNFα	GGA GAA GGG TGA CCG ACT CA	CTG CCC AGA CTC GGC AA
18s	CGG CTA CCA CAT CCA AGG AA	GCT GGA ATT ACC GCG GCT

Primer sequences (miRNA)

**miRNA of interest**	**Sequence/catalog number**	
Rat rno-miR-291b-hairpin inhibitor, QIAGEN	AAAGUGCAUCCAUUUUGUU AGU	
miRIDIAN microRNA hairpin inhibitor negative control	Cat No. IN-001005-01-05, QIAGEN	
Rn_miR-291b miScript primer assay	Cat No. MP00005292, QIAGEN	
Hs_SNORD68-11	Cat No. MS00033712, QIAGEN	
